# Education in the Transvaal: Mr. C. Louis Leipoldt's Report as Medical Inspector

**Published:** 1915-11-13

**Authors:** 


### EDUCATION IN THE TRANSVAAL.

### Mr. C. Louis Leipoldt's Report as Medical Inspector.

A most interesting report has reached us from the Medical Inspector of Schools in the Transvaal, embodying the work of his Department during the first four months of his appointment in 1914 up to the dislocation caused by the campaign against German South-West Africa. As the inspector was seconded for that section of the world war his work was interrupted for some months, and it is quite astonishing what a comprehensive survey of the subject he has been able to take and to present to his Government in so short a time and under such difficulties.

### Light and Air.

The report, as has been said, is remarkably full and thorough, and it is most interesting to trace the similarities and the differences between the problems of the school child's health as presented in South Africa and in this country. To show how different the conditions are in some respects it is sufficient to mention that "artificial light is apparently never required in the schools"; this is due, of course, partly to the clear air and strong sunshine of a high altitude, and partly to the longer (winter) day of a sub-tropical zone compared with the short, dark afternoons of our high latitude in Britain. Still it is evident that the necessity for artificial illumination was contemplated in some schools when they were built (sub-tropical thunderstorms are accompanied by a considerable degree of comparative darkness), for we read that "in many of the schools visited the electric installation was found out of order and practically useless." A few schools are defective in respect of natural lighting, but apparently the defects noted are mostly susceptible of cure. Ventilation, again, judged by European standards, is on the whole good; another result, probably, of a more kindly climate. It is noted that on the Witwatersrand, where high winds are frequent and "dust storms" are easily aroused from the by-products of the mining industry, it is "almost impossible so to construct a classroom that it shall have the maximum of light and fresh air with the minimum exposure to dust and with a minimum velocity of air entry."

### Some Structural Failings.

In the same way heating is not the problem in the Transvaal that it is with us, though the lofty Rand can be bleak enough in the (short) South African winter, as everyone who has sojourned in Johannesburg can testify. The inspector says he had no opportunity of testing the efficiency of the heating systems in the majority of cases, and seems to regard the prevailing arrangements as not uniformly satisfactory. In other respects, too, the sanitation of the school buildings appears to have had too little attention paid to it; lavatory accommodation is frequently deficient, and some severe strictures are made upon the lack of proper management and supervision of this very important section of school hygiene. Nor are the schools themselves kept clean; in only five is the school-cleaning reported to be good, while in the others the prevailing faults noted were insufficient cleaning of the furniture and fixings, littered cupboards, imperfectly cleaned corridors, dirty lavatories, and carelessly cleaned playgrounds. The playgrounds themselves, it is added, are good in the newer schools, but very faulty in many of the older ones—swamps in the wet season and exceedingly dusty and dirty in the dry one. These deficiencies are only to be expected in provinces where hitherto there has been no medical inspection, and in which only a few of the largest towns have got much beyond the corrugated-iron stage of construction, while mud and wattle is still a frequent ingredient in the building of farmhouses and homesteads on the veld. The attention directed to these important details by the medical inspector will probably lead to a higher standard in future construction and to improvements in the existing schools. Broadly speaking, the schools visited were chiefly of recent erection, and of the Pretoria and Johannesburg districts; in the smaller dorps and country towns the standard reached is probably lower. When the time comes for the inspector to visit them there may be some sensational revelations.

### Facts and Figures.

To those familiar with school medical inspection in England the statistics of defects noted and the recommendations for dealing with them may be of some interest. Since the inspections were practically confined to town children, of mixed European descent, the chief modifying factors will be climate and altitude. The figures furnished by the report amply prove that the favourable conditions which South Africa enjoys in these respects do not by any means suffice to eliminate defects, and that the proportion of children in real need of remedial treatment is quite as high as in many parts of Europe, and higher than in many places where school inspection has been systematic and thorough. Rickets and tuberculosis are considerably below the level they reach in English towns; and this is ascribed in the report to the direct sunshine so easily obtained, or, rather, so difficult to avoid, in the Transvaal. Some of the Johannesburg slums are said to be as bad as any in England, yet these two diseases are less troublesome even in the worst quarters of the town.

The inspector rightly observes that statistical tables of only one year's work would have but relative value; and avoids loading his report with columns of figures. Nevertheless, there are some percentages deducible which are perhaps worth citation. In the Witwatersrand area, out of every hundred school children, forty-one need no treatment, while fifty-nine are defective and need treatment to correct their defects. Again, in every hundred, thirty-two are clean (as regards body and head); forty-five moderately clean; twenty-three are dirty; five have bad footgear; ten are badly nourished; twenty-one are fatigued; and two are verminous—on the whole a fair showing compared with European children, but one capable of great improvement. Carious teeth are, naturally, the commonest defect, followed at a considerable interval by defects of the nose and throat and of the eye. Lower on the list still are defects of the lungs, intestinal worms, and other abdominal troubles, including chronic appendicitis. Deformities are present in 2 per cent., mental defect in 1 per cent., heart disease in 1 per cent. There is as yet no school dentist in the Transvaal, but the results of a visit by a dentist to two schools on the Rand revealed figures of dental caries which clearly show the need for such an official, as well as for the instruction of parents and children in oral prophylaxis and treatment. Seventy-one per cent. of children with more than four carious teeth and a bad general state of the mouth were thus indicated, and though the number of children inspected was small, there seems no reason to doubt that the percentage represents the general state of the school population. Clearly the Transvaal should be a dentist's paradise just now.

### Treatment.

The inspector was wise to confine his early energies to the Rand and Pretoria areas, not only because the largest number of school children were there concentrated and available for most convenient study, but also because the prejudices of those communities against inspection were likely to be less formidable than those of the back-veld farmers. He even went the length of devising schemes for treatment of the children in these areas before passing on to inspection of the country districts; and the detailed proposals put forward as a result of special meetings with representatives of the medical profession are of interest, though their translation into action was suspended by the outbreak of war.

Two centres were to be established, one for the Pretoria town and urban district and the other for the Fordsburg-Vrededorp area of Johannesburg. These clinics were to be staffed by a panel of local practitioners and dentists, the appointments to be subject to Departmental approval and to be for one year with the option of renewal. The Department (of Education) was to furnish and equip the treatment centres, to defray the working expenses, and to pay salaries to the doctors at the rate of one guinea per hour of work up to a maximum of five hours a week. The Department further undertook to provide an organiser to interview parents and to deal with the clerical and statistical work. No child was to be treated as a necessitous case where the parents were in a position to pay a private practitioner. The treatment was to be entirely in the hands of the professional staff, and a nurse was to be provided by the Department to assist the doctors at the clinic.

The report closes with certain recommendations for the future conduct and expansion of the staff, which are unexceptionable and will, it may be hoped, be acceptable to the Department and the Government. Such an excellent start has been made that the value of this school inspection must surely be self-evident; and the extension of the same system to the whole of the province is clearly imperative. The picked Afrikander, as a certain Rugby football team showed us a few years ago, is a man of stalwart frame and robustious energy. There is every reason to believe that with due attention to hygiene, nutrition, and sanitary environment in general, the wonderful climate of the South African plateau might help to breed up an Anglo-Dutch race whose average physique might almost reach that of the famous "Springbok" team. We shall want all the men and women we can get for the future of the British Empire after the war, and we shall want them healthy and strong. Intelligent support in the Transvaal of the school medical officer, Mr. C. L. Leipoldt, F.R.C.S., in whom the Government possess a most valuable servant, is well calculated to help towards that goal; and we are confident that this excellent report will result in such support being rendered.

